# Multiple sclerosis patients and COVID-19

**DOI:** 10.1186/s41983-021-00287-3

**Published:** 2021-03-23

**Authors:** Hubert Mado, Monika Adamczyk-Sowa

**Affiliations:** Department of Neurology, Faculty of Medical Sciences in Zabrze, Medical University of Silesia in Katowice, Poland, 3 Maja Street 13/15, 41-800 Zabrze, Poland

**Keywords:** Multiple sclerosis, MS, COVID-19, SARS-CoV-2, DMT

## Abstract

Coronavirus disease 2019 (COVID-19) is now a major issue for all fields of medicine. Due to the higher mortality rate among patients with chronic diseases, it has also caused concern in patients with multiple sclerosis (MS), who in addition are often receiving immunosuppressive drugs. The aim of this article is to discuss what is currently known about the severity of COVID-19 in MS patients.

## Introduction

The coronavirus disease 2019 (COVID-19) pandemic is currently a major medical topic worldwide. COVID-19 is known to have the most severe course and highest mortality in patients who are elderly and with co-morbidities such as cardiovascular diseases, chronic respiratory diseases, or diabetes [1]. Therefore, the pandemic has raised concerns about the course of COVID-19 in multiple sclerosis (MS) patients, with a particular focus on the use of disease-modifying therapies (DMTs).

## Main text

MS is a chronic disease presenting with progressive motor impairment, and MS patients, especially those with more severe forms of the disease, are on the whole more prone to infections [2]. In addition, many MS patients take DMTs, which are drugs that have immunomodulatory and also immunosuppressive effects. It is these effects of DMTs that are the main reason for fears that these drugs may be responsible for a potential more severe course of COVID-19, as well as a higher mortality rate. Of particular concern are those DMTs that lead to lymphopenia and also a reduction in the B lymphocyte count, for example cladribine, alemtuzumab, ocrelizumab, and rituximab [3–7].

However, the prevalence of COVID-19 in MS patients based on current data appears to be similar to that in the total population [3, 4]. Furthermore, the data we know so far suggest that also DMTs do not influence the severity of COVID-19 [3]. Nevertheless, there are some concerns—firstly, it seems reasonable to suspect that MS patients with significant motor impairment are likely to have a severe COVID-19 course and increased mortality due to their commonly poor general health [4]. Second, alemtuzumab and cladribine have not been widely used in previous studies, and it is suspected that DMTs causing lymphopenia and reduced B lymphocyte counts may be responsible for the increased morbidity, severity, and mortality of COVID-19 [4–7]. Noteworthy, a recently presented study in a group of 844 MS patients with suspected or confirmed COVID-19 showed that anti-CD20 therapy (rituximab or ocrelizumab) was associated with a significantly increased risk of severe COVID-19 [8]. The use of methylprednisolone in the short time before COVID-19 (less than 1 month) was further found to be a significant risk factor for severe infection [8].

## Conclusion

Thus, taking into account previous studies, recommendations, but also concerns, it seems that in the great majority of MS patients, treatment with previously used DMTs should be continued, and in the absolute majority of newly diagnosed MS patients, treatment with DMTs is to be started [9, 10]. However, therapy must be as personalized as possible and the selection of specific DTMs must consider any existing concerns with them—particularly for those with known lymphocyte-reducing effects, those causing B cell depletion, and those that have not been widely represented in COVID-19-related studies to date. In view of recent research, it seems reasonable to be extra vigilant about the use of rituximab and ocrelizumab. Also, it seems appropriate that MS patients for 1 month after methylprednisolone treatment should especially avoid any situation that could be associated with an increased risk of COVID-19 infection.

## Data Availability

Data available in a public repository that issues datasets with DOIs.

## Abbreviations

COVID-19: Coronavirus disease 2019
MS: Multiple sclerosis
DMTs: Disease-modifying therapies
